# Evaluation of Oxytetracycline Metabolites Cross-Reactivity with Oxytetracycline Enzyme-Linked Immunosorbent Assay (ELISA)

**DOI:** 10.3390/antibiotics9040183

**Published:** 2020-04-15

**Authors:** Faraj Hijaz, Nabil Killiny

**Affiliations:** Department of Plant Pathology, Citrus Research and Education Center, IFAS, University of Florida, 700 Experiment Station Road, Lake Alfred, FL 33850, USA; fhijaz@ufl.edu

**Keywords:** Huanglongbing, oxytetracycline, 4-epi-oxytetracycline, antibiotic, ELISA, citrus

## Abstract

Antibiotics have been successfully used for the control of several plant diseases for many years. Recently, streptomycin and oxytetracycline have been approved for the treatment of Huanglongbing (HLB) in Florida. The enzyme-linked immunosorbent assay (ELISA) is the most commonly used assay for the detection of these antibiotics because it is quick, simple, and can be used to analyze many samples at the same time. However, ELISA can react with the metabolites of the parent compound and its structurally related compounds. In this study, we investigated the cross-reactivity of the oxytetracycline ACCEL ELISA kit^TM^ with three of oxytetracycline metabolites (4-epi-oxytetracycline, α-apo-oxytetracycline, and β-apo-oxytetracycline). The α-apo-oxytetracycline and β-apo-oxytetracycline metabolite did not show any cross-reactivity in the linear range (1.5–50 ng mL^−1^) of the assay. Whereas 4-epi-oxytetracycline showed high cross-reactivity, and its response was similar to oxytetracycline. Our results indicated that the oxytetracycline ELISA kits estimate the level of oxytetracycline as well as its main metabolite, 4-epi-oxytetracycline.

## 1. Introduction

The citrus greening disease, Huanglongbing (HLB), is currently threatening the citrus industry in different regions of the world. In Florida, HLB is caused by *Candidatus* Liberibacter asiaticus (*C*Las) and is vectored by the Asian citrus psyllid, *Diaphorina citri*. The *D. citri* transmits the *C*Las pathogen during its feeding activities on citrus phloem sap. Currently, HLB is considered the most dangerous disease of citrus and has resulted in significant loss of citrus production in many regions. Unfortunately, most citrus cultivars are sensitive to HLB and currently, there is no cure for HLB. Control of the insect vector using insecticides is considered as the most effective tool for the control of HLB. Besides the use of insecticides, several control practices such as enhanced nutritional programs (ENPs) [1], thermotherapy [2], and removal of infected trees [3] have been suggested. However, these control practices were not effective in the field.

Due to the significant losses in the citrus industry in the last few years, the use of antibiotics was recently re-suggested for the control of HLB. The idea of using antibiotics for the control of the HLB disease was initially suggested in the 1970s after it has been discovered that HLB was caused by a microbial pathogen [2]. Previous studies showed that several antibiotics such as penicillin, ampicillin, tetracycline, and rifampicin were effective against the *C*Las pathogen [4]. In 2016, streptomycin and oxytetracycline were approved for the control of HLB disease in Florida [5].

Besides its use in agriculture, oxytetracycline is widely used in animal feeds. Because oxytetracycline has a long metabolism period, it may accumulate in high levels in meats and lead to the development of bacterial resistance [6]. Consequently, the levels of oxytetracycline and its metabolites 4-epi-oxytetracycline in meat and meat products are under strict regulation [6]. Several analytical methods have been developed to measure the level of oxytetracycline in food, including high-performance liquid chromatography (HPLC), liquid chromatography-mass spectrometry (LC-MS), chemiluminometric, and several colorimetric and fluorescence methods [6,7,8]. Although these methods have been successfully used to measure oxytetracycline in various matrixes, enzyme-linked immunosorbent assay (ELISA) is considered as the most convenient method because it is sensitive, simple, and can be used to analyze large numbers of samples simultaneously in a short time [6,9].

In our previous study, we examined the uptake, translocation, and persistence of oxytetracycline in citrus plants using ELISA [9]. Oxytetracycline was detected in the phloem, xylem, leaves, and root after stem and root treatments. Our results also showed that oxytetracycline was relatively stable in citrus plants and it was still detectable in plant tissues thirty-five days after treatment [9]. However, because the ELISA antibodies can react with oxytetracycline as well as with some of its metabolites, we decided to investigate the cross-reactivity of three of the oxytetracycline metabolites (4-epi-oxytetracycline, α-apo-oxytetracycline, and β-apo-oxytetracycline) with the antibody of the oxytetracycline ACCEL ELISA kit^TM^ (Plexense, Inc., Davis, CA, USA).

## 2. Results

**Figure 1:**
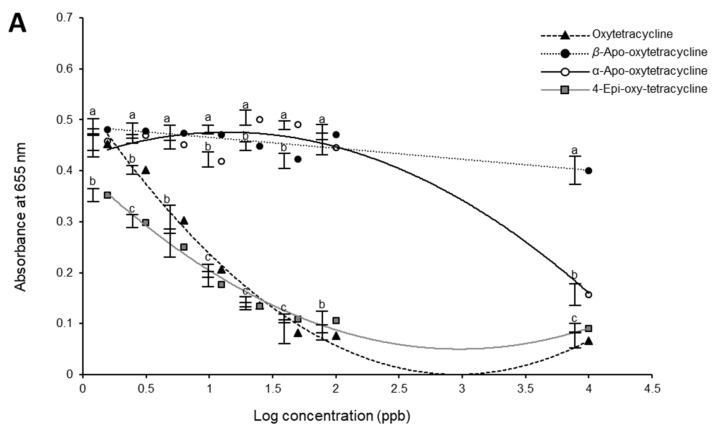

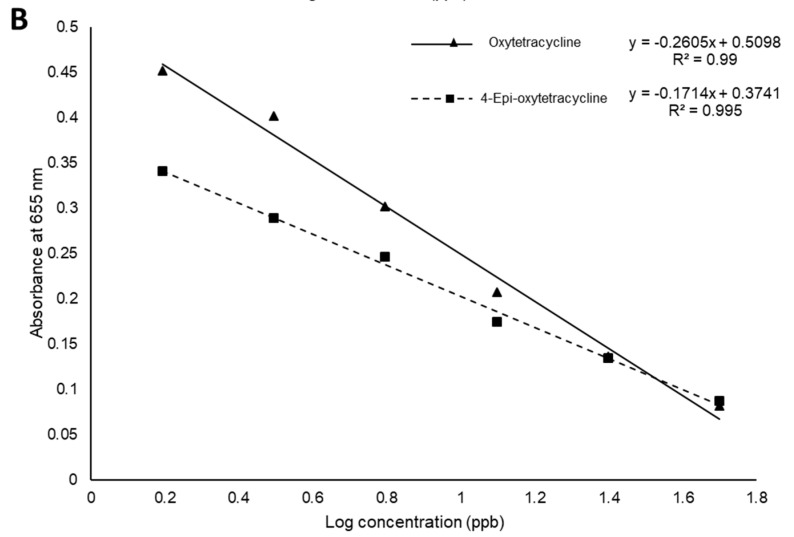
Cross-reactivity of the oxytetracycline metabolites with oxytetracycline ACCEL ELISA antibody. (**A**) The response of 4-epi-oxytetracycline, α-apo-oxytetracycline, and β-apo-oxytetracycline and oxytetracycline to oxytetracycline ACCEL ELISA antibody. The trendlines were fitted using second-order polynomial functions. (**B**) Standard curves for oxytetracycline and its main metabolite (4-epi-oxytetracycline) as generated with ACCEL ELISA kit^TM^ using the average response at each concentration in the linear range (1.56–50 ng mL^−1^). a,b,c indicate significant differences.

The response of oxytetracycline, 4-epi-oxytetracycline, α-apo-oxytetracycline, and β-apo-oxytetracycline as measured using the oxytetracycline kit is shown in Figure 1A. The α-apo-oxytetracycline and β-apo-oxytetracycline did not show any cross-reactivity with the oxytetracycline kit between 1.5–100 ng mL^−1^ (Figure 1A). The α-apo-oxytetracycline showed some cross-reactivity (65% inhibition) at a very high concentration (10,000 ng mL^−1^) (Figure 1A), which is not likely to be observed in real samples. On the other hand, 4-epi-oxytetracycline showed high cross-reactivity with the oxytetracycline antibody, and its response was similar to that of oxytetracycline (Figure 1A). The Tukey’s test showed that the response to 4-epi-oxytetracycline was similar to that of oxytetracycline, except at low concentration (1.56 and 3.13, ng mL^−1^), which were slightly lower than oxytetracycline (Figure 1). The standard curve of oxytetracycline and 4-epi-oxytetracycline in the linear range (1.56–50 ng mL^−1^) were also similar (Figure 1B).

## 3. Discussion

Our results show that the 4-epi-oxytetracycline had high cross-reactivity with the oxytetracycline ELISA antibody, whereas α-apo-oxytetracycline and β-apo-oxytetracycline did not show any cross-reactivity between 1.5–100 ng mL^−1^. In agreement with our results, Le et al. (2012) showed that 4-epi-oxytetracycline has a high cross-reactivity (98.51%) with oxytetracycline monoclonal antibody [6]. On the other hand, tetracycline, 4-epi-tetracycline, doxycycline, 4-epi-doxycycline, chlortetracycline, and 4-epi-chlortetracycline showed negligible cross-reactivity with the monoclonal antibody [6]. The high-cross reactivity of 4-epi-oxytetracycline was considered advantageous because 4-epi-oxytetracycline is the main metabolite of oxytetracycline [6].

In another study, Aga et al. (2003) evaluated the cross-reactivity of several tetracycline antibiotics and tetracycline metabolites with the tetracycline ELISA antibody (R-Biopharm GmbH, Darmstadt, Germany) [10]. The tetracycline antibodies showed to be most sensitive towards chlortetracycline, requiring only 0.21 ppb to result in a 50% reduction in the absorbance of the negative control (IC_50_). The IC_50_ of tetracycline was (1.018) ppb. The epimers and the dehydration by-products of tetracycline also showed high cross-reactivity (IC_50_; 0.3–5.3 ppb) with tetracycline antibodies [10]. Oxytetracycline also showed high cross-reactivity with the tetracycline antibody (IC_50_:0.968 ppb) [10]. The previous results suggested that slightly modified metabolites of tetracyclines and structurally related compounds are expected to give high cross-reactivity with tetracycline antibody [10]. Aga et al. (2003) warned that the ELISA might give higher results than those obtained by HPLC-MS; however this depends on the percentages of the cross-reactivity (IC_50_) of transformed metabolites or other compounds as well as the standard used in the ELISA [10].

Previous results showed that oxytetracycline breaks down to several metabolites, including 4-epi-oxytetracycline. For example, 4-epi-oxytetracycline and N-demethyloxytetracycline were detected as metabolites of oxytetracycline in egg and hen plasma [11]. In another study, 4-epi-oxytetracycline was also detected in bones (femur, breastbone, fibula, and tibia) from broilers treated with oxytetracycline [12]. In addition, several metabolites were detected as a result of abiotic degradation of oxytetracycline in the soil, including 4-epi-oxytetracycline, α-apo-oxytetracycline, and β-apo-oxytetracycline [13]. Our results showed that the oxytetracycline ACCEL ELISA kit does not only detect oxytetracycline but also detects its main metabolite, which is also under strict regulation in food [6].

## 4. Material and Methods

Oxytetracycline ACCEL ELISA kit was purchased from Plexense, Inc., (Davis, CA, USA). Oxytetracycline metabolites (4-epi-oxytetracycline, α-apo-oxytetracycline, and β-apo-oxytetracycline) were purchased from Fisher Scientific (Pittsburgh, PA, USA). Stock solutions (1 mg mL^−1^) of the oxytetracycline metabolites were prepared by dissolving 10.0 mg in 10.0 mL 0.1 N HCl. The stock solutions were diluted using the dilution buffer provided with the kit to prepare the following concentrations (10,000.0, 100.0, 50.0, 25.0, 12.50, 6.25, 3.13, 1.56 ng mL^−1^). The oxytetracycline stock solution (1 mg mL^−1^), provided with the kit, was also diluted in the same manner using the dilution buffer. The ELISA assay for oxytetracycline and its metabolites was performed according to the manufacturer’s instruction. Briefly, 120 µL of the standard was mixed with 120 µL of diluted enzyme horseradish peroxidase (HRP)-conjugate (500-fold dilution). Three mixtures were prepared for each concentration. An aliquot (100 µL) of the mixed solution was transferred into ACCEL ELISA strip and was incubated for 30 min at room temperature. At the end of the incubation time, the mixture was discarded, and the wells were washed six times with the diluted washing buffer (20-fold dilution). After discarding the washing buffer, 100 µL of the substrate solution was added and incubated for 15 min at room temperature. At the end of 15 min, the absorbance was measured using a microplate reader at 655 nm. Statistical analysis was performed using JMP 9.0 software (SAS, Cary, NC, USA). Comparison among the mean response (absorbance) of the four compounds was performed by one-way analysis of variance (ANOVA), followed by posthoc pairwise comparison using Tukey’s honestly significant difference (HSD) test (*p*-value < 0.05).

## 5. Conclusions

Our results indicated that metabolites with a similar structure to oxytetracycline are likely to bind with oxytetracycline antibodies. The metabolite, 4-epi-oxytetracycline, which is an epimer of oxytetracycline, showed high cross-reactivity, whereas α-apo-oxytetracycline, and β-apo-oxytetracycline, which are apparently different from oxytetracycline did not show any cross-reactivity. Our results showed that oxytetracycline ELISA assay results are an estimate of the total oxytetracycline and its main metabolite, 4-epi-oxytetracycline. Consequently, we believe that the use of HPLC-MS would be a better choice in order to differentiate between oxytetracycline and its metabolites.

## References

[1] Gottwald T.R., Graham J.H., Irey M.S., McCollum T.G., Wood B.W. (2012). Inconsequential effect of nutritional treatments on huanglongbing control, fruit quality, bacterial titer and disease progress. Crop. Prot..

[2] Blaustein R.A., Lorca G.L., Teplitski M. (2017). Challenges for Managing *Candidatus* Liberibacter spp. (Huanglongbing Disease Pathogen): Current Control measures and future directions. Phytopathology.

[3] Halbert S.E., Manjunath K.L. (2006). Asian citrus psyllids (sternorrhyncha: Psyllidae) and greening disease of citrus: A literature review and assessment of risk in Florida. Florida Entomol..

[4] Zhang M., Yang C., Powell C.A. (2015). Application of antibiotics for control of citrus huanglongbing. Adv. Antibiot. Antibodies.

[5] Wang N., Pierson E.A., Setubal J.C., Xu J., Levy J.G., Zhang Y., Li J., Rangel L.T., Martins J. (2017). The *Candidatus* Liberibacter–Host Interface: Insights into pathogenesis mechanisms and disease control. Annu. Rev. Phytopathol..

[6] Le T., Yu H., Zhao Z., Wei W. (2012). Development of a monoclonal antibody-based ELISA for the detection of oxytetracycline and 4-epi-oxytetracycline residues in chicken tissues. Anal. Lett..

[7] Swapna Priya S., Radha K.V. (2014). Brief review of spectrophotometric methods for the detection of tetracycline antibiotics. Int. J. Pharm. Pharm. Sci..

[8] Arnaud N., Georges J. (2001). Sensitive detection of tetracyclines using europium-sensitized fluorescence with EDTA as co-ligand and cetyltrimethylammonium chloride as surfactant. Analyst.

[9] Al-Rimawi F., Hijaz F., Nehela Y., Batuman O., Killiny N. (2019). Uptake, translocation, and stability of oxytetracycline and streptomycin in citrus plants. Antibiotics.

[10] Aga D.S., Goldfish R., Kulshrestha P. (2003). Application of ELISA in determining the fate of tetracyclines in land-applied livestock wastes. Analyst.

[11] Zurhelle G., Petz M., Mueller-Seitz E., Siewert E. (2000). Metabolites of oxytetracycline, tetracycline, and chlortetracycline and their distribution in egg white, egg yolk, and hen plasma. J. Agric. Food Chem..

[12] Cornejo J., Pokrant E., Araya D., Briceño C., Hidalgo H., Maddaleno A., Araya-Jordán C., San Martin B. (2017). Residue depletion of oxytetracycline (OTC) and 4-epi-oxytetracycline (4-epi-OTC) in broiler chicken’s claws by liquid chromatography-tandem mass spectrometry (LC-MS/MS). Food Addit. Contam. Part. A.

[13] Halling-Sørensen B., Lykkeberg A., Ingerslev F., Blackwell P., Tjørnelund J. (2003). Characterisation of the abiotic degradation pathways of oxytetracyclines in soil interstitial water using LC-MS-MS. Chemosphere.

